# The Influence of Gut Microbiota on the Cardiovascular System Under Conditions of Obesity and Chronic Stress

**DOI:** 10.1007/s11906-021-01144-7

**Published:** 2021-05-20

**Authors:** Piotr Dubinski, Katarzyna Czarzasta, Agnieszka Cudnoch-Jedrzejewska

**Affiliations:** Department of Experimental and Clinical Physiology, Laboratory of Centre for Preclinical Research, Medical University of Warsaw, Banacha 1b, 02-097 Warsaw, Poland

**Keywords:** Cardiovascular disease, Chronic stress, Gut microbiota, Heart failure, Hypertension, Obesity

## Abstract

**Purpose of Review:**

Based on the available data, it can be assumed that microbiota is an integral part of the human body. The most heavily colonized area of the human body is the gut, with bacterial accumulation ranging from 10^1^–10^3^ cells/g in the upper intestine to 10^11^–10^12^ cells/g in the colon. However, colonization of the gut is not the same throughout, as it was shown that there are differences between the composition of the microbiota in the intestine lumen and in the proximity of the mucus layer.

**Recent Findings:**

Gut microbiota gradient can be differentially regulated by factors such as obesity and chronic stress. In particular, a high fat diet influences the gut microbial composition. It was also found that chronic stress may cause the development of obesity and thus change the organization of the intestinal barrier. Recent research has shown the significant effect of intestinal microflora on cardiovascular function. Enhanced absorption of bacterial fragments, such as lipopolysaccharide (LPS), promotes the onset of “metabolic endotoxemia,” which could activate toll-like receptors, which mediates an inflammatory response and in severe cases could cause cardiovascular diseases. It is presumed that the intestinal microbiota, and especially its metabolites (LPS and trimethylamine N-oxide (TMAO)), may play an important role in the pathogenesis of arterial hypertension, atherosclerosis, and heart failure.

**Summary:**

This review focuses on how gut microbiota can change the morphological and functional activity of the cardiovascular system in the course of obesity and in conditions of chronic stress.

## Introduction

### Gut Microbiota

The available research has proven that gut microbiota is an integral part of the human body [1, 2]. Gut microbiota is a heterogeneous microbial community that contributes substantially to an open ecosystem, despite being deeply embedded within the human body. It comprises a varied and abundant microbial population consisting of bacteria, archaea, and eukaryotes that live in mutual dependence with the host [3]. A term used interchangeably for microbiota is microbiome, which strictly refers to the entire habitat, including the described microorganisms, their genomes, and the surrounding environmental conditions [4].

The most heavily colonized area of the human body is the gut, with bacterial accumulation ranging from 10^1^–10^3^ cells/g in the upper intestine to 10^11^–10^12^ cells /g in the colon [3, 5]. Due to the extremely large number of bacterial cells in the body, the host and the microorganisms inhabiting it are often referred to as a “superorganism” [2••]. It has been shown that the bacterial phyla of: *Firmicutes* (genus such as *Lactobacillus*, *Clostridium*, *Enterococcus*) and *Bacteroidetes* (genus such as *Bacteroides*) constitute the majority of gut microbiota, though other phyla such as *Actinobacteria* (*Bifidobacteria*), *Proteobacteria* (*Escherichia coli*), *Fusobacteria*, *Verrucomicrobia*, and *Archaea* are also present (Table 1) [6–10].

**Table 1:** The main representatives of the human gut microbiota, including its metabolites and location

Domain	Phylum	Genus	Metabolites	Location
Bacteria	Bacteroidetes (gram-negative bacteria)	*Bacteroides*	Propionate, succinate, LPS	Few in the stomach, 1 of the dominant in the small intestine and the colon
		*Prevotella*	Acetate, propionate, LPS	
		*Rikenella*	Propionate, LPS	
	Firmicutes (mostly gram-positive bacteria)	*Clostridium*	Acetate, butyrate, vitamin B12, TMAO	Numerous in stomach, dominant in small intestine and colon
		*Ruminococcus*	Acetate, butyrate, lactate, ethanol	
		*Faecalibacterium*	Acetate, butyrate, lactate, formate	
		*Peptostreptococcus*	Acetate, TMAO	
		*Eubacterium*	Acetate, butyrate, propionate, lactate, formate	
		*Veillonella*	Acetate, propionate	
		*Roseburia*	Acetate, butyrate, lactate, formate	
		*Bacillus*	Riboflavine (vitamin B2), vitamin B12	
		*Coprococcus*	Acetate, butyrate lactate	
		*Lactobacillus*	Vitamin B12, thiamine, pyridoxine	
		*Staphylococcus*	Lactate	
		*Streptococcus*	Acetate, vitamin B12, thiamine, pyridoxine	
	Actinobacteria (gram-positive bacteria)	*Bifidobacterium*	Acetate, folate	Mainly in stomach, sparse in colon
		*Collinsella*	Acetate, formate	
		*Actynomyces*	Acetate	
	Proteobacteria (gram-negative bacteria)	*Desulfovibrio*	Acetate, butyrate	Dominant in stomach, sparse in small intestine and colon
		*Escherichia*	Acetate, riboflavine (vitamin B2), LPS	
		*Enterobacter*	LPS	
		*Klebsiella*	LPS	
		*Proteus*	Vitamin B12	
	Fusobacteria (gram-negative bacteria)	*Fusobacterium*	Palmitoyl-sphingomyelin, p-hydroxy-benzaldehyde	Small numbers in the entire digestive tract, including colon
	Verrucomicrobia	*Akkermansia*	Acetate, propionate	Mainly present in colon
Archaea	Euryarcheota	*Methanobacter*	Methane	Duodenum, jejunum, ileum, colon

*LPS*, lipopolysaccharide; *TMAO*, trimethylamine *N*-oxide

The composition of the microbiome in the gastrointestinal (GI) tract depends on the environmental conditions prevailing in its section and is stratified both on the transverse and longitudinal axis. The bacterial cell density and composition are altered by nutritional, chemical, and immunological gradients along the gut [2••]. In the small intestine, there are generally high levels of acids, oxygen, and antimicrobials and a short passage time. Therefore, bacterial growth is limited to rapidly growing, facultative anaerobes with the ability to adhere to epithelia/mucus. Conversely, a dense and diverse bacterial community with a predominance of anaerobes, utilizing complex carbohydrates which are undigested in the small intestine, is supported by colonic conditions [2••]. There are differences between the composition of the microbiota in the intestine lumen and in the proximity of the mucus layer. For example, gram-negative *Proteobacteria* and *Akkermansia muciniphila* (phylum *Verrucomicrobia*), which use mucus as a carbon and nitrogen source, adhere and reside within the mucus layer [11].

### Positive Effects of Microbiota

**Fig. 1:**
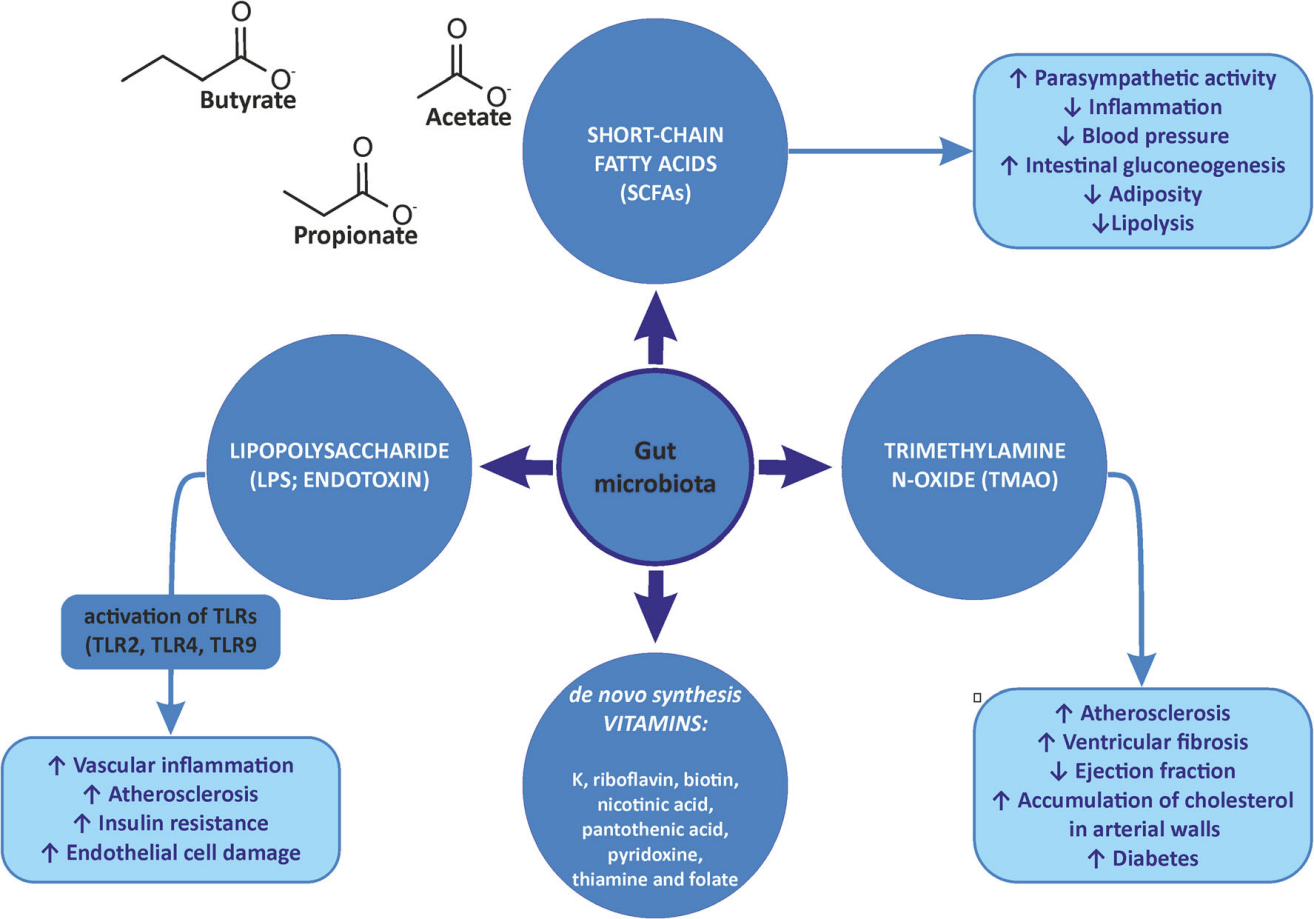
Main bacterial metabolites and their influence on the cardiovascular system. TLR, toll-like receptors

Microbiota with the correct composition and distribution in the intestines offers many positive effects to the host. First, gut microbiota synthesizes enzymes, which enables them to ferment dietary fiber to produce metabolites such as short-chain fatty acids (SCFAs) [12••]. In this way, three dominant SCFAs are formed in the intestines, i.e., acetate (C2), propionate (C3), and butyrate (C4) in the proportion 3:1:1 [2••]. Acetate is mainly produced by *Streptococcus* spp., *Prevotella* spp., *Bifidobacterium* spp., *Clostridium* spp., and *Akkermansia muciniphila*, while propionate is synthesized by *Bacteroides* spp., *Salmonella* spp., *Dialister* spp., *Veillonella* spp., *Roseburia inulinivorans*, *Coprococcus catus*, and *Blautia obeum*, and butyrate is produced by the *Lachnospiraceae*, *Ruminococcaceae*, and *Acidaminococcaceae* families (Table 1) [13–16]. These compounds can either be defecated or taken up by the gut epithelium and they impact numerous cellular processes, i.e., (1) intensify the production of interleukin-18 (IL-18), which is involved in maintaining and restoring epithelial integrity and intestinal barrier permeability, (2) prevent autoinflammation and carcinogenesis, (3) influence appetite regulation and energy intake, (4) influence hepatic lipid and glucose homeostasis, and (5) influence the differentiation of T-regulatory cells, which modulate the gut and peripheral immune responses, and maintain tolerance to self-antigens [2, 12, 16] (Fig. 1).

As already mentioned, gut microbiota influences epithelial homeostasis through regulation of mucus production and remodeling of mucin glycosylation, for example, *Lactobacilli rhamnosus* GG stimulate gut cell renewal and wound healing and *Akkermansia muciniphila* and *Lactobacillus plantarum* have been implicated in promoting epithelial integrity [2••]. In addition, microbiota impacts the contingency of other microorganisms to settle in the gut by competing for attachment sites or nutrient sources and by producing antimicrobial substances [2••]. These functions interfere with the ability of pathogens to colonize, potentially giving commensal phyla a competitive predominance in the GI tract [2, 17].

Furthermore, microbiome is essential to the de novo synthesis of vitamin K, riboflavin, biotin, nicotinic acid, pantothenic acid, pyridoxine, thiamine, and folate and takes part in the metabolism of bile acids (Table 1; Fig. 1) [2••].

### Negative Effects of Microbiota

Interactions between microbiome and a host may be altered as a result of a disrupted microbial composition, known as dysbiosis [2, 17]. In unfavorable conditions, physiological processes may be negatively affected by the excessive supply of some microbial metabolites or their increased penetration into the bloodstream [18, 19]. The first compound of this type is the gram-negative bacterial wall component lipopolysaccharide (LPS), known as endotoxin, which is involved in the initiation and progression of inflammation (Table 1; Fig. 1) [18, 20]. The innate immune system uses toll-like receptors (TLRs) to recognize LPS combined with specific proteins binding with TLRs (CD14/TLR4 complex). TLRs are a family of pattern-recognition receptors playing an essential role in innate immunity by consolidating, among other things, proinflammatory signals from microbiome–host interactions. Enhanced absorption of LPS promotes the onset of “metabolic endotoxemia,” which activates TLRs, which in turn stimulate the synthesis of various proinflammatory cytokines (interleukin 1 beta, IL-1β, and tumor necrosis factor α (TNF-α)) and cytokine-mediated cell death [18, 20]. This results in an inflammatory response and in severe cases may induce metabolic disorders such as insulin resistance and cardiovascular diseases (CVD) [18, 20, 21]. In addition, a relationship between LPS and the endocannabinoid (eCB) system is suggested. LPS stimulates eCB system tone and eCB activation stimulates adipogenesis. Therefore, LPS is considered as a significant trigger in the onset of obesity and related diseases such as type 2 diabetes [18, 20].

Moreover, intestinal microorganisms generate the organic compound trimethylamine *N*-oxide (TMAO) (Table 1; Fig. 1) [22•]. If nourishment absorption outstrips the transport capacity of the small intestine, then the nourishment reaches the colon and is metabolized by microbiota which produces trimethylamine (TMA). TMA is then further processed to TMAO by the hepatic flavin monooxygenases. The TMAO blood concentration may be modified by certain factors, including microbiome composition and diet [23]. Increased concentration of TMAO appears in the blood after ingestion of food rich in l-carnitine and choline, for example, fish, red meat, and eggs [22•]. TMAO-heightened plasma levels are involved in an increased risk of diabetes, atherosclerosis, heart fibrosis, wall thinning, and reduced ejection fraction [22, 24, 25].

It has been shown that intestinal dysbiosis can be caused, among other things, by obesity and chronic stress [26, 27].

## Gut Microbiota and Obesity

Numerous studies have proven that the imbalance in gut microbiota may pose a threat for host metabolism and energy homeostasis [18••, 28–30]. This may trigger the development of conditions such as obesity, insulin resistance and diabetes [29, 31]. Similarly, obesity predisposes to the development of dysbiosis (Table 2) [32].

**Table 2:** Gut microbiota dysbiosis in the course of HFD/obesity, chronic stress/depression, and cardiovascular disease (hypertension, atherosclerosis, heart failure)

Domain	Phylum	Genus	Dysbiosis in HFD/obesity	Dysbiosis in chronic stress/depression	Dysbiosis in cardiovascular disease		
					Hypertension	Atherosclerosis	Heart failure
**Bacteria**	**Bacteroidetes** (gram-negative bacteria)	*Bacteroides*	Decrease level [18••]	Decreased level [32–35] Increased level [36, 37]	Decreased level [38••] Increased production of butyrate—lowering blood pressure [39]	Decreased level [40]	Decreased level [41, 42]
		*Prevotella*	Decreased level [43]	Decreased level [34, 35]		Decreased level [40]	Decreased level [41, 42]
		*Rikenella*	Increased level [43]		Increased production of butyrate—lowering blood pressure [39]		Decreased level [42, 44] Increased level [41]
	**Firmicutes** (mostly gram-positive bacteria)	*Clostridium*	Increased level [43]	Increased level [32–36, 45]	Increased level [38••, 46•, 47]	Increased TMAO synthesis—proatherosclerotic effect [48]	Decreased level [42]
		*Ruminococcus*	Decreased level [43]	Decreased level [34, 35, 37] Increased level [34]		Increased level [40] Increased TMAO synthesis—proatherosclerotic effect [48]	Decreased level [49] Increased level [50]
		*Faecalibacterium*	Increased level [29]	Decreased level [34, 35, 37]		No change [40]	Decreased level [41, 42]
		*Eubacterium*	Decreased level [18••]			Decreased level [51] No change [40]	Decreased level [42]
		*Veillonella*		Increased level [34, 36]			Increased level [41, 42]
		*Roseburia*	Decreased level [18••]	Decreased level [37] Increased level [35]		Decreased level [40, 51]	
		*Bacillus*		Increased level [34, 36]			
		*Coprococcus*	Decreased level [43]	Decreased level [32, 37]	Decreased level and the production of pressure-reducing butyrate [38••, 46•]	No change [40]	
		*Lactobacillus*	Decreased level [18••, 43] Increased level [29]	Decreased level [32, 34] Increased level [34, 36]	Decreased level [47] Increase in sympathetic activity [39]	Increased level [40]	Increased level [42]
		*Staphylococcus*	Increased level [29, 43]				
		*Streptococcus*	Increased level [43]		Increased level [40, 46•] Increased production of lactate—incrising blood pressure [46•] Increase in sympathetic activity [39]	Increased level [40]	Increased level [41]
	**Actinobacteria** (gram-positive bacteria)	*Bifidobacterium*	Decreased level [18••, 29, 43]	Decreased level [34] Increased level [36, 52]	Decrease level [46•] Increase in sympathetic activity [39]	No change [40]	
		*Collinsella*		Decreased level [34] Increase level [42]		Increased level [51, 53] No change [40]	Decreased level [49]
		*Actynomyces*		Decreased level [34] Increased level [52]			
	**Proteobacteria** (gram-negative bacteria)	*Desulfovibrio*		Increased level [34]			Increased level [41, 42]
		*Escherichia*	Increased level [29]	Decreased level [35] Increased level [34]	Increase in sympathetic activity [39]	Increased level [40] Increased TMAO synthesis—proatherosclerotic effect [48]	Increased level [42, 49]
		*Enterobacter*	Increased level [29, 41]	Increased level [34, 36]		Increased level [40]	Increased level [41, 42]
		*Klebsiella*		Increased level [34]		Increased level [40]	Increased level [42]
		*Proteus*		Increased level [34]		Increased TMAO synthesis—proatherosclerotic effect [48]	Increased level [42]
	**Fusobacteria** (gram-negative bacteria)	*Fusobacterium*	Increased level [43]				
	**Verrucomicrobia**	*Verrucomicrobium*	Decreased level [43]				
		*Akkermansia*					
**Archea**	**Euryarcheota**	*Methanobacter*	Increased level [29]				

*TMAO*, trimethylamine *N*-oxide

### Obesity as a Disease

Obesity is a chronic disease considered by the World Health Organization (WHO) to be a global pandemic. There are about 2 billion adult people overweight, and of these, over 600 million are obese [54, 55]. Predominatingly, the body adiposity is assessed by body mass index (BMI), which is calculated as body weight (kg) divided by high squared (m^2^) [54, 55]. Acording to the WHO and the National Institute of Health (NIH), in adult White, Hispanic, and Black individuals, obesity is diagnosed by a BMI of 30 kg/m^2^ or greater and overweight is defined by a BMI between 25 and 29.9 kg/m^2^ [54–56]. However, BMI diagnostic value is different for men and women with similar body fatness [54, 55]. Additionally, above cut-off value of BMI is not correct for children and adolescent (age- and sex-dependent cut-off) as well as for certain ethnicities, e.g., Asian and South Asian population [54–56]. It was demonstrated that Asians have different associations between BMI, percentage of body fat, and risk of type 2 diabetes and cardiovascular disease than the Europeans [57]. Therefore, BMI cut-off point for Asian and South Asian population has been lowered: overweight is diagnosed by BMI between 23 and 24 kg/m^2^, while obesity is definied by BMI greater than 25 kg/m^2^ [56, 57].

In general, the reason for obesity can be stated as an imbalance between energy intake and its expenditure. However, the matter is much more complicated because environmental factors, alongside genetic factors affect the onset of obesity, which in itself is conducive to further dysregulation of energy management [18••, 58]. In the course of obesity, excessive adipose tissue proliferation occurs and related systemic disorders are also observed, including vascular, hormonal (insulin resistance, glucose intolerance), and systemic low-grade inflammation, leading to the development of type 2 diabetes and cardiovascular diseases such as atherosclerosis and hypertension [12••, 18••, 21, 59, 60]. It has been proven that gut microbiota can be a link between the above disorders and genetic predisposition, immunity, and environment [18••, 58].

### Correlation Between Microbiota and Obesity

Numerous experimental studies have demonstrated the influence of obesity induced in rodents by a high-fat diet (HFD) on gut microbiota, most of all by reducing the content of *Bifidobacterium* spp., *Tenericutes* spp., phylum *Bacteriodetes* and *Bacteroides* spp., *Lactobacillus* spp., *Roseburia* spp., *Eubacterium rectale* and *Blautia coccoides*, and increasing the abundance of *Firmicutes*, *Actinobacteria*, and *Proteobacteria* (Table 2) [18••, 26, 43, 61]. In addition, it was found that a change in the composition of gut microbiota in mice can support a HFD in the development of metabolic disorders such as obesity and insulin resistance [62]. Studies conducted in male Swiss albino mice and in male C57BL/6 mice on HFD show that a special role in this process is played by increased levels of *Deltaproteobacteria*, *Gammaproteobacteria*, and pathobionts (*Staphylococcus* spp., *Odoribacter* spp., *Neisseria* spp., and *Propionibacterium* spp.) [43, 61].

Similarly, it was noted in clinical studies that obesity, especially in patients with metabolic disorders, reduced the differentiation of intestine microorganisms [63]. Interestingly, a higher bacterial diversity was observed in obese patients without metabolic abnormalities than in healthy lean individuals [63]. Nevertheless, it was shown that weight reduction in obese people was well correlated with the increase in the proportion of *Bacteroidetes* over time and a fiber-enriched diet or low-fat diet can decrease the level of *Firmicutes* [64]. Moreover, pediatric, adolescent and adult studies were shown that the modyfication of the gut microflora composition by probiotics affects weight change [65, 66]. Studies conducted by Alisi et al. [67] on obese children with non-alcoholic fatty liver disease (NAFLD) showed that the administration of VSL#3, which is a mixture of eight probiotic strains (*Streptococcus thermophilus*, *Bifidobacteria* (*B. breve*, *B. infantis*, *B. longum*), *Lactobacillus acidophilu*s, *Lactobacillus plantarum*, *Lactobacillus paracasei*, and *Lactobacillus delbrueckii* subsp. *bulgaricus*), had a positive effect on BMI, fatty liver, insulin resistance, and plasma glucagon-like peptide-1 (GLP-1) concentrations. Similarly, administration of *Bifidobacterium pseudocatenulatum* CECT 7765 to obese children with insulin resistance contributed to a significant decrease in body weight [68]. However, other researchers have not confirmed the beneficial effects of probiotics on body weight in children, and it has been reported that the administration of VSL#3 to children resulted in a significant reduction in total adiposity and trunk adiposity, without significant effects on liver steatosis and liver fibrosis, gut microbial counts, or gut hormones [69]. Numerous studies on obese adults show that probiotic strains: *Lactobacillus acidophilus* LA-14, *Lactobacillus casei* LC-11, *Lactococcus lactis* LL-23, *Bifidobacterium bifidum* BB-06, *Bifidobacterium lactis* BL-4, alone or in combination, and *Pediococcus pentosaceus* contribute to a significant reduction in body weight, BMI, waist circumference, and fat mass [70–74]. However, there are also reports that contradict the above data [18••, 75]. It appears that the differences in the cited studies may result, inter alia, from the lifestyle of patients, their eating habits, and also differences between the genders. It has been reported that the administration of *Lactobacillus rhamnosus* CGMCC1.3724 together with a low calorie diet resulted in significant weight loss in obese women when compared with obese men [76]. In addition, human studies have revealed that a part of the microbiome populations is hereditary, interalia, the *Christensenellaceae* cluster, which is negatively correlated with obesity, or the phyla *Blautia* spp., which has been observed to be correlated with higher visceral fat, and *Methanobrevibacter smithii*, which has been observed to be correlated with higher BMI [58].

## Gut Microbiota and Stress

The reciprocal influence between the psychological function and various physiological functions of the digestive tract is widely discussed and has begun to be referred to as the microbiota–gut–brain axis [27•].

### Stress and Its Implications

The definition of stress indicates that it is an organism’s total response to environmental demands or pressures [27•, 77]. In general, stress can be unpredictable and uncontrollable, mild or severe, chronic, or acute [78]. In terms of health consequences, chronic stress, understood as constant stimulation and tension of the whole organism, plays a decisive role [27•]. Stress occurs in response to factors that are defined as stressors. Initially, the organism can adapt to stressors, but if intense stress persists for a long time, the risk of developing dysfunctions increases [78]. Stress symptoms affect the psyche as well as the functioning of the whole organism. The physiological stress response involves stimulation of the hormonal system and the autonomic nervous system. In particular, chronic stress results in persistent stimulation of the above systems and consequently results in elevated levels of cortisol [77]. This may lead to serious health problems including burnout and secondary conditions, e.g., depression, anxiety, cardiovascular diseases, gastrointestinal diseases, neurological diseases, musculoskeletal diseases, or diabetes [77].

### Correlation Between Microbiota and Stress

The interaction between stress and the immune system is related to the hypothalamic–pituitary–adrenal axis (HPA axis) and appears to be mediated by gut microbiota [27•]. During stress, the central nervous system response can influence gut immunity, the intestinal neuromotor function, the secretory function, and the microbiota composition. In turn, the altered microbiome may contribute to the perpetuation of inflammation and further disruption of the gut–brain communication (Table 2) [27•]. The GI tract is known to be sensitive to stress because gut microbiota can respond to the release of stress related neurochemical mediators by dysbiosis and the provision of neurochemicals. The presence of stress-related neuroendocrine catecholamines secreted by microbiota in mice has been demonstrated [79, 80]. In the mouse model of social disorders, stress-induced changes in microbiota were accompanied by changes in the level of cytokines and chemokines [32]. Similarly, other researchers in a study on male C57BL/6 mice undergoing chronic social failure (long-term exposure to the presence of larger and aggressive male CD-1 mice) showed that, in addition to behavioral disorders, a reduction in the number and diversity of the intestinal microbiome took place [33]. In the olfactory bulbectomy-induced mouse model of chronic depression, increased expression of central corticotropin-releasing factor (CRF) was associated with changes in gut microbiota [81]. Furthermore, chronic sleep deprivation (psychological stress) in male mice has been shown to increase levels of *Clostridiaceae and Lachnospiraceae* in the gastrointestinal tract [45]. Studies carried out on the mouse model have shown that chronic mild stress (CMS) affects the composition of the intestinal microflora differently depending on sex [82]. In female mice on a normal chow diet, exposure to chronic stress caused changes in the intestinal microflora becoming similar to the microbiome composition in HFD mice, while in male mice those changes were not observed [82]. Moreover, a recent experimental study has revealed that manipulation of the microbiome may modify the stress response [83]. In the course of the study, male C57BL/6J mice stressed with the chronic unpredictable mild stress protocol (CUMS) were administered a probiotic containing viable *Bifidobacterium breve* for 5 weeks. The results revealed that probiotic treatment substantially alleviated anxiety, depression, HPA axis hyperfunction and inflammation, and stress-induced dysbiosis and enhanced the SCFA levels [83]. In addition, studies indicate that stress can negatively affect the intestinal barrier homeostasis, and above all enables excessive translocation of intestinal bacteria and antigens into subepithelial tissues and contributes to inflammatory bowel disease (IBD) pathogenesis and development [84, 85]. As a consequence, dysbiosis aggravation and increasing concentration of plasma bacterial compounds (i.e., LPS and TMAO) can be expected [18••, 22•].

Clinical trials confirmed the results obtained in experimental studies. Reviews conducted on patients with irritable bowel syndrome (IBS), which very often develops as a result of chronic stress, showed a reduction in the *Bacteroides* spp., *Parabacteroides* spp., *Prevotella* spp., and *Veillonella* spp. population and an increased *Lactobacillus* spp., *Bacillus* spp., *Bifidobacterium* spp., *Clostridiales*, and *Eubacterium rectale* population when compared with healthy volunteers [36, 52, 76, 86].

## Influence of Gut Microbiota on Hypertension

Hypertension is defined as an office-measured systolic blood pressure (SBP) of ≥ 140 mmHg and/or a diastolic blood pressure (DBP) of ≥ 90 mmHg. In 2015, the number of people with hypertension worldwide was 1.13 billion. It is estimated that by 2025, the number of people with hypertension will increase to 1.5 billion [87].

The evidence for the important role of intestinal microflora in the pathogenesis of hypertension is provided by experimental studies conducted especially on rats with spontaneous arterial hypertension (SHR) and on their natural control—WKY rats. It was found that SHR rats had a fivefold higher ratio of *Firmicutes* to *Bacterioidetes* at the phylum level, while the *Actinobacteria* and *Bifidobacterium* populations at the genus level decreased compared with WKY rats [46•]. In addition, a linear discriminant analysis effect size (LEfSe) study showed that lactate-producing bacteria such as *Streptococcus* spp. and *Turicibacter* spp. were predominant in SHR rats, whereas in WKY rats, butyrate-producing bacteria were predominant, including *Coprococcus* spp. and *Pseudobutyrivibrio* spp. [46•]. The influence of intestinal microflora on arterial hypertension has also been confirmed by recent studies by Toral et al. [38••] in which fecal microflora taken from donors (WKY rats and/or SHR rats) was transplanted into the recipients (WKY rats and/or SHR rats). These studies showed that intestinal bacteria can modify the gut–brain communication and, as a result, change blood pressure. These researchers observed significantly higher values of initial systolic and diastolic blood pressure in WKY rats that were given fecal microflora taken from SHR rats (W–S) [38••]. Similarly, in the deoxycorticosterone acetate (DOCA)–salt mouse model, fiber supplementation increased the number of acetate-producing bacteria and decreased dysbiosis as measured by the ratio of *Firmicutes* to *Bacteroidetes*, which positively correlated with a decrease in SBP and DBP (Table 2) [88].

### Influence of Gut Microbiota on Hypertension in the Course of Obesity

An experimental study on pigs with metabolic syndrome (MetS) has shown gut dysbiosis, accompanied by the development of hypertension, obesity, hyperlipidemia, and insulin resistance [89•]. Moreover, gut dysbiosis in pigs with MetS was similar to the composition of gut microflora observed in human patients with MetS. Namely, pigs with MetS contained increased abundances of proinflammatory bacteria and secondary bile acid-producing bacteria, as well as a decreased population of enteroprotective bacteria and SCFAs-producing bacteria [89•].

Similarly, clinical studies conducted on the Colombian adult community have revealed that higher SCFAs levels in feces were positively correlated with fewer intestinal bacteria, higher intestinal permeability, hypertension, generalized inflammation, obesity, and dyslipidemia [12••]. In addition, studies carried out on overweight and obese pregnant women in the 16th week of pregnancy demonstrated that SBP and DBP were positively correlated with BMI and negatively correlated with an abundance of specific butyrate-producing phyla in gut microbiota including *Odoribacteraceae* and *Clostridiaceae* [39].

### Influence of Gut Microbiota on Hypertension in the Course of Stress

Evidence of the effect of stress on disturbances in the composition of microbiota and its different effects on the cardiovascular system is provided by studies of chronic prenatal stress (PNS) in 4-month-old male Sprague Dawley rats whose mothers were subjected to chronic immobilization stress during late pregnancy (from embryonic day 14 to day 20) [47]. It has been demonstrated that induced PNS decreased the numbers of bacteria in the *Lactobacillus* genus, accompanied by elevated abundance of three genera in different families of the Clostridiales order: *Oscillibacter*, *Anaerotruncus*, and *Peptococcus* genera [47]. Disorders of intestinal microflora were correlated with a higher response to stress on the HPA axis, as well as altered respiratory control, impairment of cognitive function, and elevation of blood pressure [47]. Special attention has also recently been given to a new mechanism of hypertension in which the cooperation of the intestines, brain, and bones plays a key role [90]. Rodent studies indicate increased intestinal sympathetic activity driven by stress as an implicit cause of dysbiosis, enteritis, and increased gut barrier permeability, which in turn leads to an imbalance in gut SCFAs and plasma LPS concentrations [38••, 90]. The above substances play an important role in increasing sympathetic innervation of the lymphoid organs including bone marrow, and may stimulate the proliferation and release of proinflammatory cells, particularly myeloid progenitors. This leads to the development of generalized inflammation, which is believed to be a risk factor for hypertension [90, 91]. Studies by Toral et al. [38••] showed that fecal microflora taken from SHR rats and transplanted into WKY rats causes intestinal dysbiosis and cause inducing systemic inflammation, accompanied by microglia activation and oxidative stress, leading to neuroinflammation in the paraventricular nucleus (PVN) [38••]. Neuroinflammation was identified as a significant component of neurogenic hypertension genesis [46•]. Therefore, microbiota as an inflammatory status regulator has been suggested as being able to influence the brain’s cardiovascular control areas (such as the PVN) involved in regulating blood pressure [38••, 46•].

### Influence of Gut Microbiota on Atherosclerosis

Atherosclerosis is a chronic inflammatory disease in which there is an excessive accumulation of lipids and inflammatory cells in the inner layer (tunica intima) of the arteries [92, 93]. Based on the literature, it may be assumed that intestinal microbiota plays an important role in the pathogenesis of atherosclerosis by modulating inflammation and the production of microbial metabolites (Table 2) [94]. Numerous experimental studies have shown that, in particular, TMAO plays an important role in the development of atherosclerosis, possibly due to the reduction of HDL and phospholipid levels in plasma as well as increasing the accumulation of cholesterol by macrophages and the formation of foam cells (Table 2) [22•, 24, 48, 95].

Clinical trials in patients with atherosclerosis showed a lower number of the genus of *Roseburia* and *Eubacterium* and a higher number of the genus of *Collinsella* compared with healthy controls [51]. It has also been found that some bacteria, e.g., *Akkermansia muciniphila*, can improve the intestinal barrier function and exert a protective effect against atherosclerosis [96].

### Influence of Gut Microbiota on Atherosclerosis in the Course of Obesity

Intestinal microbiota is currently regarded as being able to influence host metabolism and contribute to the development of obesity with accompanying metabolic endotoxemia and associated diseases such as atherosclerosis [18••, 97•]. In the course of obesity, metabolic disorders develop, including hypercholesterolemia, which is a common form of hyperlipidemia [98]. It has been demonstrated that in hyperlipidemic conditions, macrophages accumulate in the blood vessel walls and there they facilitate lipid uptake from the blood stream, leading to the formation of foam cells, which are a component of atheromatous plaques [97•]. These macrophages have been shown to have a proinflammatory profile induced by TLRs, which bind microbial molecules such as LPS [18••, 97•]. Research by Chen et al. [99] performed on ApoE KO mice, an animal model of atherosclerosis, showed that intestinal microbiota under hyperlipidemic conditions resulted in the recruitment and ectopic activation of B2 cells (subtype of B cells) in the perivascular adipose tissue. This was followed by an increase in circulating immunoglobulin G (IgG), which directly changed the morphology of the blood vessels, facilitating the formation of atherosclerotic plaque and accelerating the development of atherosclerosis [99].

### Influence of Gut Microbiota on Atherosclerosis in the Course of Stress

It has been proven that homeostasis disturbances in the bidirectional gut–brain axis, in conditions of chronic stress or dysbiosis, increase the risk of neuropsychiatric diseases (i.e., anxiety and depression), neurovascular diseases (i.e., cerebral atherosclerosis and ischemic stroke) and cardiometabolic diseases (i.e., atherosclerosis, obesity, diabetes) [40, 100]. However, only a few studies describe the impact of specific phyla disproportions in the course of gut dysbiosis caused by chronic stress on the development of atherosclerosis. Maes et al. [101] showed that the prevalences and median values for serum IgM and IgA against LPS of *Enterobacteria* (*Hafnia alvei*, *Pseudomonas aeruginosa*, *Morganella morganii*, *Pseudomonas putida*, *Citrobacter koseri*, *Klebsiella pneumonia*) were significanty greater in patients with major depression disorder (MDD) than in healthy volunteers. It appears that LPS translocation occurring as a result of intestinal mucosal dysfunction (leaky gut) observed during dysbiosis caused by chronic stress, plays a significant role in the inflammatory pathophysiology of depression and atherosclerosis [18••, 101].

## Influence of Gut Microbiota on Heart Failure

According to the 2016 ESC guidelines for the diagnosis and treatment of acute and chronic heart failure, heart failure (HF) is a set of typical symptoms (e.g., dyspnea, edema of the lower limbs, decreased exercise tolerance), which may be accompanied by abnormalities in physical examinations (e.g., dilatation of the jugular veins, crackle above the lungs, peripheral edema), caused by abnormalities in the structure and/or function of the heart resulting in decreased cardiac output and/or increased intracardiac pressure at rest or during exercise [102]. It is estimated that HF affects 1%–2% of the adult population in developed countries [102].

Numerous clinical studies have shown a significant influence of intestinal microflora on the development of HF (Table 2) [42, 49, 103, 104]. Heart failure patients presented intestinal dysbiosis in the form of a relative reduction in taxa from the *Lachnospiraceae* and *Ruminococcaceae* families, known for their capacity for butyrate production [42, 49, 50]. Diminished proportions of butyrate-producing gut microbiota have been associated with intestinal and extra-intestinal disorders, such as IBD, and also obesity, diabetes mellitus, and CVD [42]. Moreover, clinical studies have shown an increase in the concentration of TMAO levels in the blood of patients with HF [104–107].

### Influence of Gut Microbiota on Heart Failure in the Course of Obesity

A few studies indicate a relationship between gut dysbiosis and obesity in the pathogenesis of HF. Battson et al. [108•] has shown that cecal microbiota transplantation (CMT) from obese leptin-deficient (Ob) mice with ischemia/reperfusion myocardial infarction to C57BL/6J control (Con) mice with ischemia/reperfusion myocardial infarction caused an increased myocardial infarct size and an increased left ventricular mass as well as arterial stiffness, which were associated with greater gut permeability and reduced concentrations of cecal SCFAs, whereas in the other direction cecal microbiota transplantation (CMT) from Con mice to Ob mice resulted in a reduced myocardial infarct size and a reduced left ventricular mass as well as higher levels of cecal SCFAs [108•].

Moreover, clinical studies also appear to confirm the positive correlation between obesity-related dysbiosis and HF. Patients with coronary artery disease (CAD) and type 2 diabetes presented with significant lower abundance of phylum *Bacteroidetes*, and higher phyla *Firmicutes* and *Proteobacteria*. Futhermore, these patients had significantly less beneficial or commensal bacteria (such as *Faecalibacterium prausnitzii* and *Bacteroides fragilis*) and more opportunistic pathogens (such as *Enterobacteriaceae*, *Streptococcus*, and *Desulfovibrio*) (Table 2) [41]. The above dysbiosis can lead to increased TMAO plasma concentrations which in turn could affect the development of HF [22•, 25, 44]. This is probably due to the influence of TMAO on the reduction of beta-oxidation of fatty acids in cardiomyocytes, that leads to an excessive accumulation of fatty acids in the myocardium, which has a lipotoxic effect and leads to cardiomyocyte apoptosis [109].

### Influence of Gut Microbiota on Heart Failure in the Course of Stress

Stress leads to increased permeability of the gut allowing microorganisms and their antigens to cross the epithelial barrier and induce a mucosal immune response. Chronic stress enables the persistence of such conditions which in turn alters the composition of the microbiome and leads to enhanced activation of the HPA axis [110]. Impaired HPA axis tone precipitates the development of heart failure associated with myocardial infarction, left-ventricular dysfunction, and dysrhythmia [111].

Clinical evidence has shown that chronic stress in the form of neuropsychiatric disorders contributes to the development and progression of heart failure. Prevalent illnesses in patients with heart failure are depression and anxiety disorders (i.e., generalized anxiety disorder (GAD), post-traumatic stress disorder (PTSD), and panic disorder) which increase the risk of death or cardiac events [112].

## Conclusions

Gut microbiota is an integral part of the human body and affects the function of the human body. Factors such as obesity and chronic stress lead to dysbiosis, contributing to the development of diseases including cardiovascular, hypertension (in particular), atherosclerosis, and heart failure. Therefore, it appears to be very important to maintain the integrity of the human microbiome. Recently, attention has also been given to the therapeutic aspect of gut microbiota. However, knowledge about the interaction of gut microbiota and the human body, especially in conditions of obesity and stress, is still relatively small. Consequently, further research is needed to understand how to maintain homeostasis between the human body environment and the microbiome that inhabits it.

## References

[1] Nicholson JK, Holmes E, Wilson ID (2005). Gut microorganisms, mammalian metabolism and personalized health care. Nat Rev Microbiol..

[2] •• Thursby E, Juge N. Introduction to the human gut microbiota. Biochem J. 2017;474:1823–36. 10.1042/BCJ20160510. **This paper contain key information about composition of human gut microbiota.** 10.1042/BCJ20160510 PMC5433529 28512250

[3] Derrien M, van Hylckama Vlieg JET (2015). Fate, activity, and impact of ingested bacteria within the human gut microbiota. Trends Microbiol..

[4] Marchesi JR, Ravel J (2015). The vocabulary of microbiome research: a proposal. Microbiome..

[5] O’Hara AM, Shanahan F (2006). The gut flora as a forgotten organ. EMBO Rep..

[6] Bang C, Schmitz RA (2015). Archaea associated with human surfaces: not to be underestimated. FEMS Microbiol Rev..

[7] Dethlefsen L, McFall-Ngai M, Relman DA (2007). An ecological and evolutionary perspective on human-microbe mutualism and disease. Nature..

[8] Eckburg PB, Bik EM, Bernstein CN, Purdom E, Dethlefsen L, Sargent M (2005). Diversity of the human intestinal microbial flora. Science..

[9] Geerlings SY, Kostopoulos I, de Vos WM, Belzer C (2018). Akkermansia muciniphila in the human gastrointestinal tract: when, where, and how?. Microorganisms..

[10] Qin J, Li R, Raes J, Arumugam M, Burgdorf KS, Manichanh C (2010). A human gut microbial gene catalogue established by metagenomic sequencing. Nature..

[11] van Passel MW, Kant R, Zoetendal EG, Plugge CM, Derrien M, Malfatti SA (2011). The genome of Akkermansia muciniphila, a dedicated intestinal mucin degrader, and its use in exploring intestinal metagenomes. PLoS One..

[12] •• de la Cuesta-Zuluaga J, Mueller NT, Álvarez-Quintero R, Velásquez-Mejía EP, Sierra JA, Corrales-Agudelo V, et al. Higher Fecal Short-chain fatty acid levels are associated with gut microbiome dysbiosis, obesity, hypertension and cardiometabolic disease risk factors. nutrients. Nutrients. 2018;11:51. 10.3390/nu11010051. **This ppaer suggest the role of gut microbiom dysbiosis in obesity, hypertension and cardiometabolic disease**. 10.3390/nu11010051 PMC6356834 30591685

[13] Duncan SH, Barcenilla A, Stewart CS, Pryde SE, Flint HJ (2002). Acetate utilization and butyryl coenzyme A (CoA):acetate-CoA transferase in butyrate-producing bacteria from the human large intestine. Appl Environ Microbiol..

[14] Louis P, Flint HJ (2017). Formation of propionate and butyrate by the human colonic microbiota. Environ Microbiol..

[15] Ma J, Li H (2018). The role of gut microbiota in atherosclerosis and hypertension. Front Pharmacol..

[16] Overby HB, Ferguson JF (2021). Gut microbiota-derived short-chain fatty acids facilitate microbiota:host cross talk and modulate obesity and hypertension. Curr Hypertens Rep..

[17] Kaysen A, Heintz-Buschart A, Muller EEL, Narayanasamy S, Wampach L, Laczny CC (2017). Integrated meta-omic analyses of the gastrointestinal tract microbiome in patients undergoing allogeneic hematopoietic stem cell transplantation. Transl Res..

[18] •• Cani PD, Osto M, Geurts L, Everard A. Involvement of gut microbiota in the development of low-grade inflammation and type 2 diabetes associated with obesity. Gut Microbes. 2012;3:279–88. 10.4161/gmic.19625. **This paper provides evidences of the role of gut microbiota in pathogenesis of type 2 diabetes and obesity.** 10.4161/gmic.19625 PMC3463487 22572877

[19] Sinha R, Ahn J, Sampson JN, Shi J, Yu G, Xiong X (2016). Fecal microbiota, fecal metabolome, and colorectal cancer interrelations. PLoS One..

[20] Kell DB, Pretorius E (2015). On the translocation of bacteria and their lipopolysaccharides between blood and peripheral locations in chronic, inflammatory diseases: the central roles of LPS and LPS-induced cell death. Integr Biol (Camb).

[21] Katsimichas T, Antonopoulos AS, Katsimichas A, Ohtani T, Sakata Y, Tousoulis D (2019). The intestinal microbiota and cardiovascular disease. Cardiovasc Res..

[22] • Leustean AM, Ciocoiu M, Sava A, Costea CF, Floria M, Tarniceriu CC, et al. Implications of the intestinal microbiota in diagnosing the progression of diabetes and the presence of cardiovascular complications. J Diabetes Res. 2018;2018:5205126. 10.1155/2018/5205126. **The data from this paper releveals implications of the intestinal microbiota in diagnosis the progression of diabetes and the presence of cardiovascular complications.** 10.1155/2018/5205126 PMC6260408 30539026

[23] Koeth RA, Wang Z, Levison BS, Buffa JA, Org E, Sheehy BT (2013). Intestinal microbiota metabolism of L-carnitine, a nutrient in red meat, promotes atherosclerosis. Nat Med..

[24] Lüscher TF (2021). They eat, what we eat, they digest, what we ingest: the microbiome and the vulnerable plaque. Cardiovasc Res..

[25] Warmbrunn MV, Herrema H, Aron-Wisnewsky J, Soeters MR, Van Raalte DH, Nieuwdorp M (2020). Gut microbiota: a promising target against cardiometabolic diseases. Expert Rev Endocrinol Metab..

[26] Cândido FG, Valente FX, Grześkowiak ŁM, Moreira APB, Rocha DMUP, Alfenas RCG (2018). Impact of dietary fat on gut microbiota and low-grade systemic inflammation: mechanisms and clinical implications on obesity. Int J Food Sci Nutr..

[27] • De Palma G, Collins SM, Bercik P, Verdu EF. The microbiota-gut-brain axis in gastrointestinal disorders: stressed bugs, stressed brain or both? J Physiol. 2014;592:2989–97. 10.1113/jphysiol.2014.273995. T**his paper suggest the role of microbiota-gut-brain axis in gastrointestinal disorders.** 10.1113/jphysiol.2014.273995 PMC4214655 24756641

[28] Clemente JC, Ursell LK, Parfrey LW, Knight R (2012). The impact of the gut microbiota on human health: an integrative view. Cell..

[29] Million M, Lagier JC, Yahav D, Paul M (2013). Gut bacterial microbiota and obesity. Clin Microbiol Infect..

[30] Musso G, Gambino R, Cassader M (2010). Gut microbiota as a regulator of energy homeostasis and ectopic fat deposition: mechanisms and implications for metabolic disorders. Curr Opin Lipidol..

[31] Baothman OA, Zamzami MA, Taher I, Abubaker J, Abu-Farha M (2016). The role of gut microbiota in the development of obesity and diabetes. Lipids Health Dis..

[32] Bailey MT, Dowd SE, Galley JD, Hufnagle AR, Allen RG, Lyte M (2011). Exposure to a social stressor alters the structure of the intestinal microbiota: implications for stressor-induced immunomodulation. Brain Behav Immun..

[33] Bharwani A, Mian MF, Foster JA, Surette MG, Bienenstock J, Forsythe P (2016). Structural & functional consequences of chronic psychosocial stress on the microbiome & host. Psychoneuroendocrinology..

[34] Chong PP, Chin VK, Looi CY, Wong WF, Madhavan P, Yong VC. The microbiome and Iirritable bowel syndrome - A review on the pathophysiology, Current research and future therapy. front microbiol. 2019;10:1136. 10.3389/fmicb.2019.01136. 10.3389/fmicb.2019.01136 PMC6579922 31244784

[35] Jiang H, Ling Z, Zhang Y, Mao H, Ma Z, Yin Y, et al. Altered fecal microbiota composition in patients with major depressive disorder. Brain Behav Immun. 2015;48:186–94. 10.1016/j.bbi.2015.03.016 10.1016/j.bbi.2015.03.016 25882912

[36] Maccaferri S, Candela M, Turroni S, Centanni M, Severgnini M, Consolandi C (2012). IBS-associated phylogenetic unbalances of the intestinal microbiota are not reverted by probiotic supplementation. Gut Microbes..

[37] Hu S, Li A, Huang T, Lai J, Li J, Sublette ME, et al. Gut Microbiota changes in patients with bipolar depression. Adv Sci (Weinh). 2019;6:1900752. 10.1002/advs.201900752. 10.1002/advs.201900752 PMC6662053 31380217

[38] •• Toral M, Robles-Vera I, de la Visitación N, Romero M, Yang T, Sánchez M, et al. Critical role of the interaction gut microbiota - sympathetic nervous system in the regulation of blood pressure. Front Physiol. 2019;10:231. 10.3389/fphys.2019.00231. **This paper describes the role of the interaction gut microbiota and sympathetic nervous syetm in the regulation of blood pressure**. 10.3389/fphys.2019.00231 PMC6423906 30930793

[39] Gomez-Arango LF, Barrett HL, McIntyre HD, Callaway LK, Morrison M, Dekker Nitert M (2016). SPRING Trial Group. Increased systolic and diastolic blood pressure is associated with altered gut microbiota composition and butyrate production in early pregnancy. Hypertension..

[40] Jie Z, Xia H, Zhong SL, Feng Q, Li S, Liang S (2017). The gut microbiome in atherosclerotic cardiovascular disease. Nat Commun..

[41] Sanchez-Alcoholado L, Castellano-Castillo D, Jordán-Martínez L, Moreno-Indias I, Cardila-Cruz P, Elena D (2017). Role of gut microbiota on cardio-metabolic parameters and immunity in coronary artery disease patients with and without type-2 diabetes mellitus. Front Microbiol..

[42] Kamo T, Akazawa H, Suda W, Saga-Kamo A, Shimizu Y, Yagi H (2017). Dysbiosis and compositional alterations with aging in the gut microbiota of patients with heart failure. PLoS One..

[43] Singh DP, Singh J, Boparai RK, Zhu J, Mantri S, Khare P (2017). Isomalto-oligosaccharides, a prebiotic, functionally augment green tea effects against high fat diet-induced metabolic alterations via preventing gut dysbacteriosis in mice. Pharmacol Res..

[44] Dambrova M, Latkovskis G, Kuka J, Strele I, Konrade I, Grinberga S (2016). Diabetes is associated with higher trimethylamine N-oxide plasma levels. Exp Clin Endocrinol Diabetes..

[45] El Aidy S, Bolsius YG, Raven F, Havekes R (2019). A brief period of sleep deprivation leads to subtle changes in mouse gut microbiota. J Sleep Res..

[46] • Yang T, Santisteban MM, Rodriguez V, Li E, Ahmari N, Carvajal JM, et al. Gut dysbiosis is linked to hypertension. Hypertension. 2015;65:1331–40. 10.1161/HYPERTENSIONAHA.115.05315. **This paper suggest the role of gut dysbiosis in the pathogenesis of hypertension**. 10.1161/HYPERTENSIONAHA.115.05315 PMC4433416 25870193

[47] Golubeva AV, Crampton S, Desbonnet L, Edge D, O’Sullivan O, Lomasney KW (2015). Prenatal stress-induced alterations in major physiological systems correlate with gut microbiota composition in adulthood. Psychoneuroendocrinology..

[48] Wang Z, Roberts AB, Buffa JA, Levison BS, Zhu W, Org E (2015). Non-lethal Inhibition of gut microbial trimethylamine production for the treatment of atherosclerosis. Cell..

[49] Luedde M, Winkler T, Heinsen FA, Rühlemann MC, Spehlmann ME, Bajrovic A (2017). Heart failure is associated with depletion of core intestinal microbiota. ESC Heart Fail..

[50] Cui X, Ye L, Li J, Jin L, Wang W, Li S (2018). Metagenomic and metabolomic analyses unveil dysbiosis of gut microbiota in chronic heart failure patients. Sci Rep..

[51] Karlsson FH, Fåk F, Nookaew I, Tremaroli V, Fagerberg B, Petranovic D (2012). Symptomatic atherosclerosis is associated with an altered gut metagenome. Nat Commun..

[52] Jeffery IB, O’Toole PW, Öhman L, Claesson MJ, Deane J, Quigley EM, et al. An irritable bowel syndrome subtype defined by species-specific alterations in faecal microbiota. Gut. 2012;61:997–1006. 10.1136/gutjnl-2011-301501. 10.1136/gutjnl-2011-301501 22180058

[53] Morera LP, Marchiori GN, Medrano LA, Defagó MD (2019). Stress, dietary patterns and cardiovascular disease: a mini-review. Front Neurosci..

[54] Durrer Schutz D, Busetto L, Dicker D, Farpour-Lambert N, Pryke R, Toplak H (2019). European practical and patient-centred guidelines for adult obesity management in primary care. Obes Facts..

[55] Yumuk V, Tsigos C, Fried M, Schindler K, Busetto L, Micic D, et al. Obesity Management Task Force of the European Association for the Study of Obesity. European guidelines for obesity management in adults. Obes Facts. 2015;8:402–24. 10.1159/000442721. 10.1159/000442721 PMC5644856 26641646

[56] Weir CB, Jan A. BMI classification percentile and cut off points. In: StatPearls. Treasure Island (FL): StatPearls Publishing; 2021. https://www-1ncbi-1nlm-1nih-1gov-100001ati0892.han3.wum.edu.pl/books/NBK541070/Accessed Jan. 2020 Jul 10. 31082114

[57] Expert WHO (2004). Consultation. Appropriate body-mass index for Asian populations and its implications for policy and intervention strategies. Lancet..

[58] Chang CS, Ruan JW, Kao CY (2019). An overview of microbiome based strategies on anti-obesity. Kaohsiung J Med Sci..

[59] Luck H, Khan S, Kim JH, Copeland JK, Revelo XS, Tsai S (2019). Gut-associated IgA + immune cells regulate obesity-related insulin resistance. Nat Commun..

[60] Tawakol A, Ishai A, Takx RA, Figueroa AL, Ali A, Kaiser Y (2017). Relation between resting amygdalar activity and cardiovascular events: a longitudinal and cohort study. Lancet..

[61] Velázquez KT, Enos RT, Bader JE, Sougiannis AT, Carson MS, Chatzistamou I (2019). Prolonged high-fat-diet feeding promotes non-alcoholic fatty liver disease and alters gut microbiota in mice. World J Hepatol..

[62] Garidou L, Pomié C, Klopp P, Waget A, Charpentier J, Aloulou M (2015). The gut microbiota regulates intestinal CD4 T cells expressing RORγt and controls metabolic disease. Cell Metab..

[63] Zeng Q, Li D, He Y, Li Y, Yang Z, Zhao X (2019). Discrepant gut microbiota markers for the classification of obesity-related metabolic abnormalities. Sci Rep..

[64] Ley RE, Turnbaugh PJ, Klein S, Gordon JI (2006). Microbial ecology: human gut microbes associated with obesity. Nature..

[65] Abenavoli L, Scarpellini E, Colica C, Boccuto L, Salehi B, Sharifi-Rad J (2019). Gut microbiota and obesity: a role for probiotics. Nutrients..

[66] Cerdó T, García-Santos JA, Bermúdez MG, Campoy C (2019). The role of probiotics and prebiotics in the prevention and treatment of obesity. Nutrients..

[67] Alisi A, Bedogni G, Baviera G, Giorgio V, Porro E, Paris C (2014). Randomised clinical trial: the beneficial effects of VSL#3 in obese children with non-alcoholic steatohepatitis. Aliment Pharmacol Ther..

[68] Sanchis-Chordà J, Del Pulgar EMG, Carrasco-Luna J, Benítez-Páez A, Sanz Y, Codoñer-Franch P (2019). Bifidobacterium pseudocatenulatum CECT 7765 supplementation improves inflammatory status in insulin-resistant obese children. Eur J Nutr..

[69] Jones RB, Alderete TL, Martin AA, Geary BA, Hwang DH, Palmer SL, et al. Probiotic supplementation increases obesity with no detectable effects on liver fat or gut microbiota in obese Hispanic adolescents: a 16-week, randomized, placebo-controlled trial. Pediatr Obes. 2018;13:705–14. 10.1111/ijpo.12273. 10.1111/ijpo.12273 PMC6113106 29493105

[70] Gomes AC, de Sousa RG, Botelho PB, Gomes TL, Prada PO, Mota JF (2017). The additional effects of a probiotic mix on abdominal adiposity and antioxidant status: a double-blind, randomized trial. Obesity (Silver Spring).

[71] Higashikawa F, Noda M, Awaya T, Danshiitsoodol N, Matoba Y, Kumagai T, et al. Antiobesity effect of Pediococcus pentosaceus LP28 on overweight subjects: a randomized, double-blind, placebo-controlled clinical trial. Eur J Clin Nutr. 2016;70:582–7. 10.1038/ejcn.2016.17. 10.1038/ejcn.2016.17 26956126

[72] Kim J, Yun JM, Kim MK, Kwon O, Cho B (2018). Lactobacillus gasseri BNR17 supplementation reduces the visceral fat accumulation and waist circumference in obese adults: a randomized, double-blind, placebo-controlled trial. J Med Food..

[73] Minami J, Iwabuchi N, Tanaka M, Yamauchi K, Xiao JZ, Abe F, et al. Effects of Bifidobacterium breve B-3 on body fat reductions in pre-obese adults: a randomized, double-blind, placebo-controlled trial. Biosci Microbiota Food Health. 2018;37:67–75. 10.12938/bmfh.18-001. 10.12938/bmfh.18-001 PMC6081611 30094122

[74] Pedret A, Valls RM, Calderón-Pérez L, Llauradó E, Companys J, Pla-Pagà L (2019). Effects of daily consumption of the probiotic Bifidobacterium animalis subsp. lactis CECT 8145 on anthropometric adiposity biomarkers in abdominally obese subjects: a randomized controlled trial. Int J Obes (Lond).

[75] Borgeraas H, Johnson LK, Skattebu J, Hertel JK, Hjelmesaeth J (2018). Effects of probiotics on body weight, body mass index, fat mass and fat percentage in subjects with overweight or obesity: a systematic review and meta-analysis of randomized controlled trials. Obes Rev..

[76] Sanchez M, Darimont C, Panahi S, Drapeau V, Marette A, Taylor VH (2017). Effects of a diet-based weight-reducing program with probiotic supplementation on satiety efficiency, eating behaviour traits, and psychosocial behaviours in obese individuals. Nutrients..

[77] Anghelescu IG, Edwards D, Seifritz E, Kasper S (2018). Stress management and the role of Rhodiola rosea: a review. Int J Psychiatry Clin Pract..

[78] Lucassen PJ, Pruessner J, Sousa N, Almeida OF, Van Dam AM, Rajkowska G (2014). Neuropathology of stress. Acta Neuropathol..

[79] Lyte M, Vulchanova L, Brown DR (2011). Stress at the intestinal surface: catecholamines and mucosa-bacteria interactions. Cell Tissue Res..

[80] Sudo N (2019). Biogenic Amines: signals between commensal microbiota and gut physiology. Front Endocrinol (Lausanne).

[81] Park AJ, Collins J, Blennerhassett PA, Ghia JE, Verdu EF, Bercik P, et al. Altered colonic function and microbiota profile in a mouse model of chronic depression. Neurogastroenterol Motil. 2013;25:733–e575. 10.1111/nmo.12153. 10.1111/nmo.12153 PMC3912902 23773726

[82] Bridgewater LC, Zhang C, Wu Y, Hu W, Zhang Q, Wang J (2017). Gender-based differences in host behavior and gut microbiota composition in response to high fat diet and stress in a mouse model. Sci Rep..

[83] Tian P, O’Riordan KJ, Lee YK, Wang G, Zhao J, Zhang H (2020). Towards a psychobiotic therapy for depression: Bifidobacterium breve CCFM1025 reverses chronic stress-induced depressive symptoms and gut microbial abnormalities in mice. Neurobiol Stress..

[84] Boudry G, Jury J, Yang PC, Perdue MH (2007). Chronic psychological stress alters epithelial cell turn-over in rat ileum. Am J Physiol Gastrointest Liver Physiol..

[85] Lennon EM, Maharshak N, Elloumi H, Borst L, Plevy SE, Moeser AJ (2013). Early life stress triggers persistent colonic barrier dysfunction and exacerbates colitis in adult IL-10-/- mice. Inflamm Bowel Dis..

[86] Noor SO, Ridgway K, Scovell L, Kemsley EK, Lund EK, Jamieson C (2010). Ulcerative colitis and irritable bowel patients exhibit distinct abnormalities of the gut microbiota. BMC Gastroenterol..

[87] Williams B, Mancia G, Spiering W, Agabiti Rosei E, Azizi M, Burnier M (2018). ESC Scientific Document Group. 2018 ESC/ESH Guidelines for the management of arterial hypertension. Eur Heart J..

[88] Marques FZ, Nelson E, Chu PY, Horlock D, Fiedler A, Ziemann M (2017). High-fiber diet and acetate supplementation change the gut microbiota and prevent the development of hypertension and heart failure in hypertensive mice. Circulation..

[89] • O’Donovan AN, Herisson FM, Fouhy F, Ryan PM, Whelan D, Johnson CN, et al. Gut microbiome of a porcine model of metabolic syndrome and HF-pEF. Am J Physiol Heart Circ Physiol. 2020;318:H590–603. 10.1152/ajpheart.00512.2019. **The data from this paper reveals the role of gut microbiom in the development of metabolic syndrom and chronic heart failure**. 10.1152/ajpheart.00512.2019 32031871

[90] Santisteban MM, Kim S, Pepine CJ, Raizada MK (2016). Brain-gut-bone marrow axis: implications for hypertension and related therapeutics. Circ Res..

[91] Santisteban MM, Ahmari N, Carvajal JM, Zingler MB, Qi Y, Kim S (2015). Involvement of bone marrow cells and neuroinflammation in hypertension. Circ Res..

[92] Fioranelli M, Bottaccioli AG, Bottaccioli F, Bianchi M, Rovesti M, Roccia MG (2018). Stress and inflammation in coronary artery disease: a review psychoneuroendocrineimmunology-based. Front Immunol..

[93] Landmesser U, Chapman MJ, Farnier M, Gencer B, Gielen S, Hovingh GK (2017). European Society of Cardiology (ESC); European Atherosclerosis Society (EAS). European Society of Cardiology/European Atherosclerosis Society Task Force consensus statement on proprotein convertase subtilisin/kexin type 9 inhibitors: practical guidance for use in patients at very high cardiovascular risk. Eur Heart J..

[94] Kasahara K, Tanoue T, Yamashita T, Yodoi K, Matsumoto T, Emoto T (2017). Commensal bacteria at the crossroad between cholesterol homeostasis and chronic inflammation in atherosclerosis. J Lipid Res..

[95] Wang Z, Klipfell E, Bennett BJ, Koeth R, Levison BS, Dugar B (2011). Gut flora metabolism of phosphatidylcholine promotes cardiovascular disease. Nature..

[96] Li J, Lin S, Vanhoutte PM, Woo CW, Xu A (2016). Akkermansia muciniphila protects against atherosclerosis by preventing metabolic endotoxemia-induced inflammation in Apoe-/- mice. Circulation..

[97] • Caesar R, Fåk F, Bäckhed F. Effects of gut microbiota on obesity and atherosclerosis via modulation of inflammation and lipid metabolism. J Intern Med. 2010;268:320–8. 10.1111/j.1365-2796.2010.02270.x. **This paper suggest the role of gut microbiota in the pathogenesis of the atherosclerosis**. 10.1111/j.1365-2796.2010.02270.x 21050286

[98] Csonka C, Sárközy M, Pipicz M, Dux L, Csont T (2016). Modulation of hypercholesterolemia-induced oxidative/nitrative stress in the heart. Oxid Med Cell Longev..

[99] Chen L, Ishigami T, Nakashima-Sasaki R, Kino T, Doi H, Minegishi S, et al. Commensal microbe-specific activation of B2 cell subsets contributes to atherosclerosis development independently of lipid metabolism. EBioMedicine. 2016;13:237–47. 10.1016/j.ebiom.2016.10.030. 10.1016/j.ebiom.2016.10.030 PMC5264349 27810309

[100] Zhu S, Jiang Y, Xu K, Cui M, Ye W, Zhao G (2020). The progress of gut microbiome research related to brain disorders. J Neuroinflammation..

[101] Maes M, Kubera M, Leunis JC (2008). The gut-brain barrier in major depression: intestinal mucosal dysfunction with an increased translocation of LPS from gram negative enterobacteria (leaky gut) plays a role in the inflammatory pathophysiology of depression. Neuro Endocrinol Lett..

[102] Ponikowski P, Voors AA, Anker SD, Bueno H, Cleland JGF, Coats AJS (2016). ESC Scientific Document Group. 2016 ESC Guidelines for the diagnosis and treatment of acute and chronic heart failure: The Task Force for the diagnosis and treatment of acute and chronic heart failure of the European Society of Cardiology (ESC)Developed with the special contribution of the Heart Failure Association (HFA) of the ESC. Eur Heart J..

[103] Sata Y, Marques FZ, Kaye DM (2020). The emerging role of gut dysbiosis in cardio-metabolic risk factors for heart failure. Curr Hypertens Rep..

[104] Trøseid M, Andersen GØ, Broch K, Hov JR (2020). The gut microbiome in coronary artery disease and heart failure: Current knowledge and future directions. EBioMedicine..

[105] Trøseid M, Ueland T, Hov JR, Svardal A, Gregersen I, Dahl CP (2015). Microbiota-dependent metabolite trimethylamine-N-oxide is associated with disease severity and survival of patients with chronic heart failure. J Intern Med..

[106] Suzuki T, Heaney LM, Bhandari SS, Jones DJ, Ng LL (2016). Trimethylamine N-oxide and prognosis in acute heart failure. Heart..

[107] Tang WH, Wang Z, Fan Y, Levison B, Hazen JE, Donahue LM (2014). Prognostic value of elevated levels of intestinal microbe-generated metabolite trimethylamine-N-oxide in patients with heart failure: refining the gut hypothesis. J Am Coll Cardiol..

[108] • Battson ML, Lee DM, Li Puma LC, Ecton KE, Thomas KN, Febvre HP, et al. Gut microbiota regulates cardiac ischemic tolerance and aortic stiffness in obesity. Am J Physiol Heart Circ Physiol. 2019;317:H1210–20. 10.1152/ajpheart.00346.2019. **This paper suggest the role of gut microbiota in obesity and cardiovascular disease.** 10.1152/ajpheart.00346.2019 PMC6960779 31559829

[109] Makrecka-Kuka M, Volska K, Antone U, Vilskersts R, Grinberga S, Bandere D (2017). Trimethylamine N-oxide impairs pyruvate and fatty acid oxidation in cardiac mitochondria. Toxicol Lett..

[110] Dinan TG, Cryan JF (2012). Regulation of the stress response by the gut microbiota: implications for psychoneuroendocrinology. Psychoneuroendocrinology..

[111] Brotman DJ, Golden SH, Wittstein IS (2007). The cardiovascular toll of stress. Lancet..

[112] Celano CM, Villegas AC, Albanese AM, Gaggin HK, Huffman JC (2018). Depression and anxiety in heart failure: a review. Harv Rev Psychiatry..

